# Roles of the mitochondrial Na^+^-Ca^2+^ exchanger, NCLX, in B lymphocyte chemotaxis

**DOI:** 10.1038/srep28378

**Published:** 2016-06-22

**Authors:** Bongju Kim, Ayako Takeuchi, Masaki Hikida, Satoshi Matsuoka

**Affiliations:** 1 Center for Innovation in Immunoregulative Technology and Therapeutics, Graduate School of Medicine, Kyoto University, Yoshida-konoe, Sakyo-ku, Kyoto 606-8501, Japan; 2 Department of Integrative and Systems Physiology, Faculty of Medical Sciences, University of Fukui, 23-3 Matsuokashimoaizuki, Eiheiji-cho, Yoshida-gun, Fukui 910-1193, Japan

## Abstract

Lymphocyte chemotaxis plays important roles in immunological reactions, although the mechanism of its regulation is still unclear. We found that the cytosolic Na^+^-dependent mitochondrial Ca^2+^ efflux transporter, NCLX, regulates B lymphocyte chemotaxis. Inhibiting or silencing NCLX in A20 and DT40 B lymphocytes markedly increased random migration and suppressed the chemotactic response to CXCL12. In contrast to control cells, cytosolic Ca^2+^ was higher and was not increased further by CXCL12 in NCLX-knockdown A20 B lymphocytes. Chelating intracellular Ca^2+^ with BAPTA-AM disturbed CXCL12-induced chemotaxis, suggesting that modulation of cytosolic Ca^2+^ via NCLX, and thereby Rac1 activation and F-actin polymerization, is essential for B lymphocyte motility and chemotaxis. Mitochondrial polarization, which is necessary for directional movement, was unaltered in NCLX-knockdown cells, although CXCL12 application failed to induce enhancement of mitochondrial polarization, in contrast to control cells. Mouse spleen B lymphocytes were similar to the cell lines, in that pharmacological inhibition of NCLX by CGP-37157 diminished CXCL12-induced chemotaxis. Unexpectedly, spleen T lymphocyte chemotaxis was unaffected by CGP-37157 treatment, indicating that NCLX-mediated regulation of chemotaxis is B lymphocyte-specific, and mitochondria-endoplasmic reticulum Ca^2+^ dynamics are more important in B lymphocytes than in T lymphocytes. We conclude that NCLX is pivotal for B lymphocyte motility and chemotaxis.

Migration of lymphocytes is one of the fundamental processes of the immune response. In the case of B lymphocytes, circulating naive lymphocytes enter lymph nodes through high endothelial venules, migrate to the B cell area where chemoattractants such as CXCL12 and CXCL13 are highly expressed, and are then activated and differentiated into plasma cells^1^,^2^. This directional movement of the cell toward chemoattractants, i.e. chemotaxis, is triggered by an interaction between chemokines and their receptors. The interaction of a chemokine with its receptor (e.g. CXCL12 with CXCR4, and CXCL13 with CXCR5), as well as the subsequent signal transduction pathways, such as MAPKs, PI3K, and NF-κB, have been extensively studied using a variety of cell types, including lymphocytes (see reviews^3^,^4^,^5^,^6^). However, detailed mechanisms underlying lymphocyte chemotaxis have not yet been clarified.

Ca^2+^ is an important second messenger which regulates chemotaxis. It is well known that various cellular processes involved in cell motility are Ca^2+^-sensitive. For example, a rise or oscillation in cytosolic Ca^2+^ activates cytoskeletal remodelling through the small GTPase Rac1, thereby modulating migration of T lymphocytes, tumour mast cells and podocytes^7^,^8^,^9^. The Ca^2+^ mobilization is mediated by Ca^2+^ influx via plasma membrane Ca^2+^ channels and/or Ca^2+^ release from intracellular stores such as the endoplasmic reticulum (ER). Various Ca^2+^ carriers localized at the plasma membrane and in the ER, such as transient receptor potential channels (TRPs), the store-operated Ca^2+^ entry system (stim1/orai1), ER Ca^2+^ release channels (inositol trisphosphate receptors), and the ER Ca^2+^ pump (SERCA), have been implicated in the regulation of the migration of various kinds of cells^10^,^11^,^12^,^13^,^14^. As for T lymphocytes, several lines of evidence suggest the involvement of TRPs and stim1/orai1 in Ca^2+^ mobilization-mediated migration, though little information is available on B lymphocyte migration^11^,^12^.

Besides the ER, mitochondria provide an important Ca^2+^ store inside the cell. Mitochondrial Ca^2+^ dynamics is determined by influx via the Ca^2+^ uniporter and efflux via the Na^+^-Ca^2+^ exchanger and/or the H^+^-Ca^2+^ exchanger^15^,^16^. In 2013, MCU, which encodes the mitochondrial Ca^2+^ uniporter, and MICU, which is a regulator of MCU, were reported to be involved in the migration of human endothelial cells and zebrafish blastomeres^17^,^18^. MCU-mediated alterations in cytosolic Ca^2+^ dynamics and/or the production of mitochondrial reactive oxygen species were implicated in migration, though the exact mechanisms are still unresolved. Moreover, little is known as to whether, and if so, how mitochondrial Ca^2+^ efflux transporters participate in lymphocyte chemotaxis.

We recently demonstrated that NCLX, a gene responsible for the mitochondrial Na^+^-Ca^2+^ exchanger^19^, acts to provide Ca^2+^ to the endoplasmic/sarcoplasmic reticulum (ER/SR), and that NCLX-mediated Ca^2+^ recycling between mitochondria and the ER/SR modulates various cellular functions^20^,^21^,^22^. In B lymphocytes, NCLX is pivotal in maintaining cellular Ca^2+^ responses to antigens^20^,^21^. Considering that cytosolic Ca^2+^ dynamics participate in the migration of various kinds of cells, we hypothesized that NCLX may participate in lymphocyte migration and/or chemotaxis. In the present study, we studied the roles of NCLX in lymphocyte chemotaxis. We found that NCLX reduction/inhibition markedly suppressed chemotaxis while increasing the random migration of B lymphocytes. In addition, we found that the contributions of NCLX to motility were observed only in B lymphocytes and not in T lymphocytes.

## Results

### Facilitation of random migration and inhibition of chemotaxis by NCLX silencing in B lymphocytes

**Figure 1:**
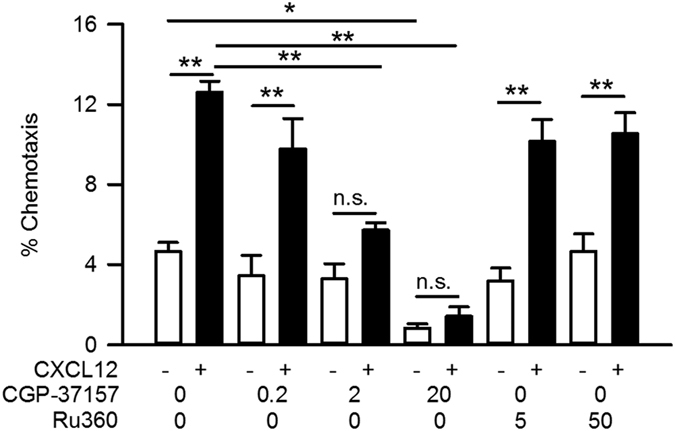
Suppression of chemotaxis by mitochondrial Na^+^-Ca^2+^ exchange inhibition in A20 B lymphocytes. Effects of a mitochondrial Na^+^-Ca^2+^ exchange inhibitor (CGP-37157) and a mitochondrial Ca^2+^ uniporter inhibitor (Ru360) on CXCL12-induced chemotaxis in a Transwell assay. Cells were pretreated with various concentrations of drugs as indicated (μM) and then were applied to the upper chamber. CXCL12 (100 ng/ml) was applied to the lower chamber in the presence or absence of the drug. N = 4. Data are expressed as mean ± SEM. **P < 0.01, *P < 0.05, n.s. not significant.

We first investigated whether pharmacological intervention of mitochondrial Ca^2+^ carriers affected the chemotaxis of A20 B lymphocytes. A standard Transwell assay was employed to measure chemotaxis. When CXCL12 was present in the bottom chamber, there was a significant increase in the movement of A20 B lymphocytes toward the bottom chamber; that is, CXCL12 induced chemotaxis of A20 B lymphocytes (Fig. 1). Perturbation of mitochondrial Na^+^-Ca^2+^ exchange by CGP-37157 (IC_50_ = 0.36 μM)^23^ dose-dependently decreased the extent of chemotaxis, while it did not affect the basal cell movement up to 2 μM. A high concentration of CGP-37157 (20 μM) significantly decreased both chemotaxis and basal cell movement. On the other hand, inhibition of the mitochondrial Ca^2+^ uniporter by Ru360 (IC_50_ = 0.2–2 nM)^24^,^25^ did not affect the extent of chemotaxis or basal cell movement, even at 50 μM (Fig. 1).

We then tested whether the mitochondrial Na^+^-Ca^2+^ exchanger-mediated modulation of chemotaxis occurred in another B lymphocyte cell line, DT40. In heterozygous NCLX knockout DT40 B lymphocytes (NCLX^+/−^), in which NCLX protein expression was negligible^20^, CXCL12-induced chemotaxis was significantly suppressed (Supplementary Fig. S1), suggesting that the mitochondrial Na^+^-Ca^2+^ exchanger is a key determinant for the chemotaxis of B lymphocytes.

**Figure 2:**
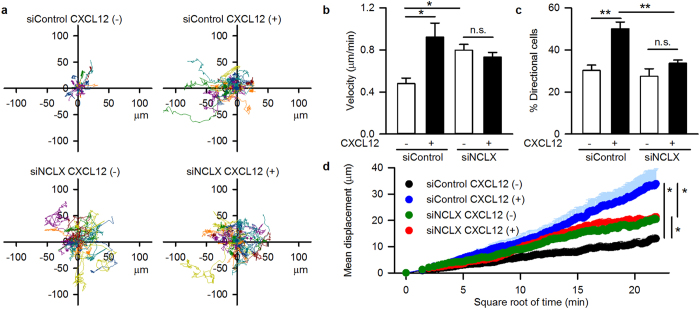
Facilitation of random migration and suppression of chemotaxis by NCLX knockdown in A20 B lymphocytes. Effects of NCLX knockdown on CXCL12-induced chemotaxis in a real-time chemotaxis assay. N = 4–5. (**a**) Representative data for the trajectory of each cell movement. CXCL12 (100 ng/ml) was applied to the left side of the reservoir. (**b**) Velocity of cells. (**c**) Percentage of cells which moved toward CXCL12. (**d**) Mean displacement of cells. Data are expressed as mean ± SEM. siControl, control siRNA transfected cells. siNCLX, NCLX siRNA transfected cells. **P < 0.01, *P < 0.05, n.s. not significant.

To evaluate individual cell motility, we next performed live-cell imaging using A20 B lymphocytes. Silencing of NCLX using siRNA has previously been shown to reduce NCLX mRNA expression^20^. We reconfirmed this in the present study by performing quantitative PCR, which resulted in the reduction of NCLX mRNA expression to 45.4 ± 10.8% (N = 3). Single-cell tracking revealed that control siRNA-transfected A20 B lymphocytes (siControl) moved randomly in the absence of CXCL12. Interestingly, under this condition, NCLX knockdown (siNCLX) cells showed higher velocity and mean displacement (Fig. 2a,b,d), indicating that NCLX knockdown facilitated random migration. In siControl cells, a CXCL12 gradient increased motility and induced directional movement toward CXCL12; chemotaxis clearly occurred (Fig. 2a–d). On the contrary, in the siNCLX cells, the CXCL12 gradient did not further increase motility or induce directional movement toward CXCL12 (Fig. 2a–d); this is comparable to the results of the Transwell assays. Similar results were obtained using 2 μM CGP-37157 to block mitochondrial Na^+^-Ca^2+^ exchange in A20 B lymphocytes (Supplementary Fig. S2a–c). In addition, random cell movement and migration velocity were higher in NCLX^+/−^ DT40 B lymphocytes compared with that in wild-type (WT) cells (Supplementary Fig. S2d,e), in accord with the facilitation of migration by the reduction or inhibition of NCLX in A20 B lymphocytes (Fig. 2a,b,d, Supplementary Fig. S2). Bath application of CXCL12 did not further augment the random cell movement and the migration velocity of NCLX^+/−^ DT40 B lymphocytes (Supplementary Fig. S2d,e); this is comparable to the results obtained from A20 B lymphocytes (Fig. 2, Supplementary Fig. S2). We confirmed that the expression level of the CXCL12 receptor, CXCR4, was unaffected by NCLX knockdown (Supplementary Fig. S3), suggesting that NCLX reduction/inhibition-mediated alterations of motility were not due to altered CXCL12/CXCR4 interaction. Thus, the silencing of NCLX facilitated random migration while inhibiting chemotaxis in B lymphocytes.

### Association of altered cytosolic Ca^2+^ with NCLX-mediated modulation of cell motility

**Figure 3:**
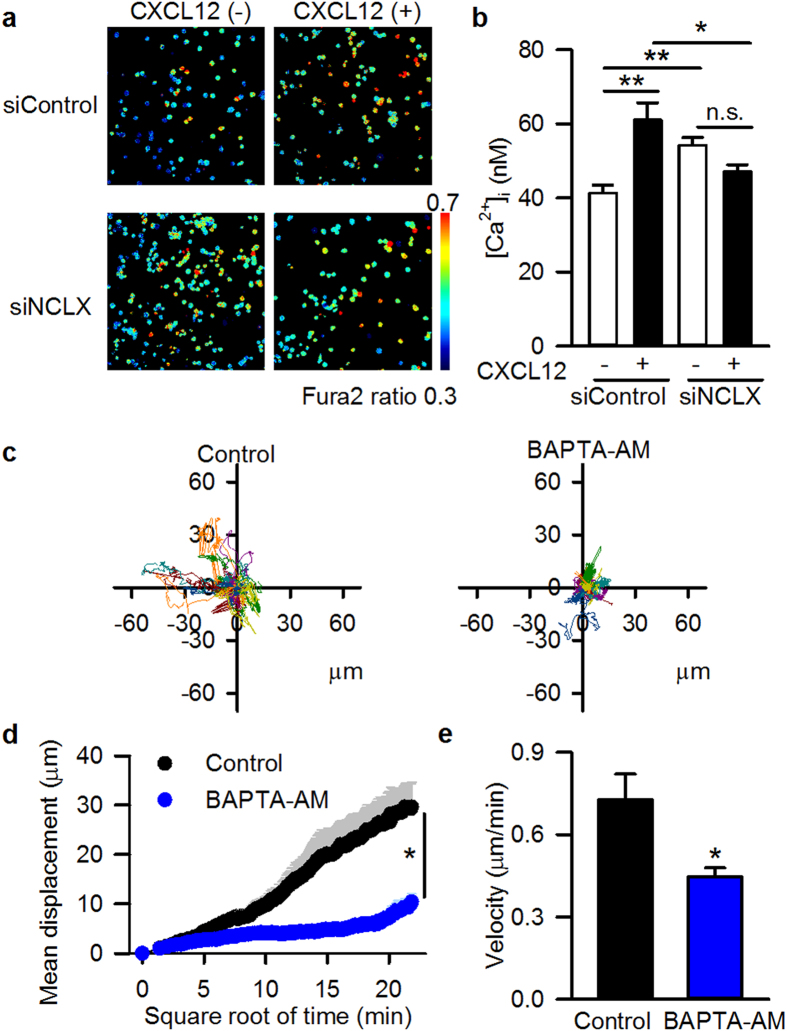
Importance of cytosolic Ca^2+^ for the motility of A20 B lymphocytes. (**a**,**b**) Effects of NCLX knockdown on cytosolic Ca^2+^ in the absence and presence of 100 ng/ml CXCL12, evaluated by staining the cells with Fura 2-AM (5 μM). **(a)** Representative data 2 hrs after the CXCL12 application. (**b**) Summary. N = 6. (**c–e**) Effects of chelating cytosolic Ca^2+^ on CXCL12-induced chemotaxis. (**c**) Representative data for cell trajectory. CXCL12 (100 ng/ml) was applied to the left side of the reservoir, and chemotaxis was observed in the absence (left) or presence (right) of the Ca^2+^ chelator BAPTA-AM (25 μM) in both sides of the reservoir. (**d**) Mean displacement of cells. (**e**) Velocity of cells. N = 4. Data are expressed as mean ± SEM. **P < 0.01, *P < 0.05, n.s. not significant.

Considering that various cellular processes involved in motility are Ca^2+^ sensitive^10^,^11^,^12^,^13^,^14^, and that NCLX is pivotal for cytosolic Ca^2+^ signalling during B cell receptor activation^20^, we hypothesized that NCLX participates in motility via modulation of cytosolic Ca^2+^. In siNCLX cells, cytosolic Ca^2+^ concentration was higher than that in siControl cells in the absence of CXCL12 (Fig. 3a,b). In siControl cells, CXCL12 treatment significantly increased the cytosolic Ca^2+^. However, it did not further increase the cytosolic Ca^2+^ in siNCLX cells (Fig. 3a,b). Essentially the same results were obtained by blocking mitochondrial Na^+^-Ca^2+^ exchange using CGP-37157 at 2 and 20 μM; i.e. treatment with CGP-37157 increased cytosolic Ca^2+^ in the absence of CXCL12, and CXCL12 application did not further increase but rather decreased cytosolic Ca^2+^ when the cells were treated with CGP-37157 (Supplementary Fig. S4). These results well correspond to the data showing that siNCLX cells had higher motility than siControl cells in the absence of CXCL12, and that CXCL12 application induced chemotaxis in siControl cells, but not in siNCLX cells (Fig. 2a,b,d). The importance of cytosolic Ca^2+^ in cell motility was confirmed by chelating cytosolic Ca^2+^ during a live-cell chemotaxis assay. Treatment of the cells with 25 μM BAPTA-AM significantly diminished motility, as revealed by decreased mean displacement as well as decreased velocity (Fig. 3c–e). The above findings suggested that altered cytosolic Ca^2+^ was associated with NCLX-mediated modulation of cell motility.

### Facilitation of F-actin polymerization and Rac1 localization in NCLX knockdown cells

**Figure 4:**
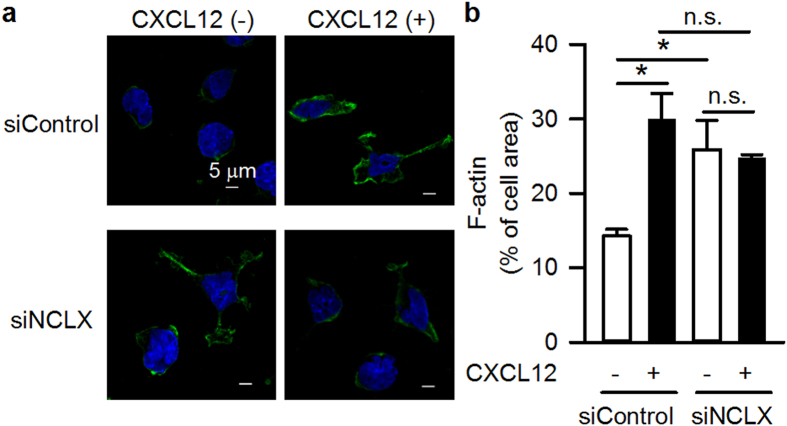
Augmentation of F-actin polymerization in NCLX knockdown A20 B lymphocytes. Effects of NCLX knockdown on F-actin polymerization in the absence and presence of 100 ng/ml CXCL12, evaluated by using fluorescently labelled phalloidin (green). Nuclei were stained with DAPI (blue). (**a**) Representative data. (**b**) Summary. N = 3. Data are expressed as mean ± SEM. *P < 0.05, n.s. not significant.

In order to elucidate how cytosolic Ca^2+^ alteration modulates cell motility in NCLX-silenced cells, we next focused on actin rearrangement and localization of Rac1, a member of a small GTPase family, because both had been reported to be Ca^2+^-sensitive and to be key factors determining cell migration and chemotaxis^8^,^9^,^26^,^27^,^28^,^29^,^30^,^31^. The distribution of F-actin was evaluated by staining the cells with fluorescently-labelled phalloidin. In siControl cells, F-actin was confined to a small area in the absence of CXCL12. On the other hand, siNCLX cells had a less confined F-actin distribution than siControl cells. After bath application of CXCL12, the F-actin region was significantly expanded in siControl cells but was unaltered in siNCLX cells (Fig. 4). Localization of Rac1 was examined by immunocytochemistry. As clearly shown in Supplementary Fig. S5, Rac1 was more diffusely distributed in siNCLX cells than in siControl cells in the absence of CXCL12. After bath application of CXCL12, the Rac1 region expanded in siControl cells but contracted in siNCLX cells. The effects of NCLX knockdown and/or CXCL12 application on F-actin and Rac1 localization were quite similar in pattern to the effects on cytosolic Ca^2+^ and cell motility. Accordingly, it was suggested that the NCLX knockdown-mediated increase of cytosolic Ca^2+^ facilitated F-actin formation and Rac1 localization, resulting in the augmentation of random cell migration.

### Association of CXCL12-induced polarization of mitochondria with directional cell movement of A20 B lymphocytes

**Figure 5:**
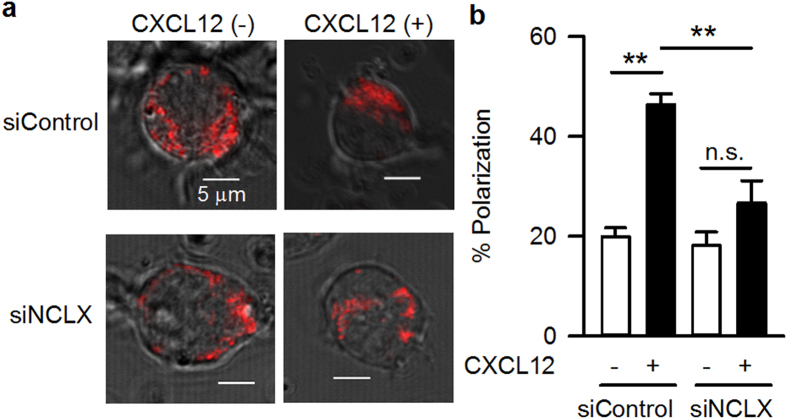
Attenuation of CXCL12-induced mitochondrial polarization in NCLX knockdown A20 B lymphocytes. Effects of NCLX knockdown on mitochondrial polarization in the absence or presence of 100 ng/ml CXCL12, evaluated by staining the cells with a mitochondria specific dye (MitoTracker Orange, red). (**a**) Representative data. (**b**) Summary. N = 15. Data are expressed as mean ± SEM. **P < 0.01, *P < 0.05, n.s. not significant.

At this point, we were faced with the question of why directional movement towards CXCL12, i.e. chemotaxis, did not occur in siNCLX cells, in spite of the increased random cell migration. In seeking the answer, we analysed mitochondrial polarization (Fig. 5), which had been reported to be important for directional cell movement in a variety of cell types^32^,^33^,^34^,^35^. In siControl cells, mitochondria stained with MitoTracker Orange were distributed evenly within the cell, and CXCL12 application induced mitochondrial accumulation in one part of the cell. In siNCLX cells, the mitochondrial distribution was similar to siControl cells in the absence of CXCL12, despite the alteration of F-actin formation and Rac1 localization (Fig. 4 and Supplementary Fig. S5). CXCL12 application failed to induce mitochondrial polarization in the siNCLX cells. Thus, the CXCL12-induced mitochondrial polarization may be associated with the directional movement of the cell and chemotaxis but not with velocity and extent of movement. Similar results were obtained using NCLX^+/−^ DT40 B lymphocytes (Supplementary Fig. S6).

### B lymphocyte specificity of NCLX-mediated modulation of chemotaxis

**Figure 6:**
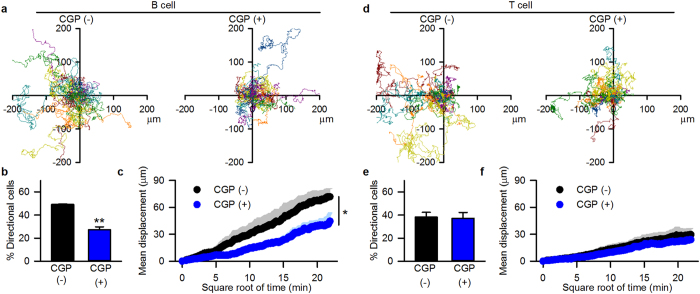
NCLX-mediated modulation of chemotaxis in spleen B lymphocytes. Effects of the mitochondrial Na^+^-Ca^2+^ exchange inhibitor CGP-37157 on CXCL12-induced chemotaxis in mouse spleen B lymphocytes (**a–c**) and T lymphocytes (**d–f**). (**a**,**d**) Representative data for cell trajectory. CXCL12 (100 ng/ml) was applied to the left side of the reservoir, and chemotaxis was observed in the absence or presence of CGP-37157 (2 μM) in both sides of the reservoir. (**b**,**e**) Percentage of cells which moved toward CXCL12. (**c**,**f**) Mean displacement of cells. N = 3–4. Data are expressed as mean ± SEM. **P < 0.01, *P < *0.05.*

Finally, we examined whether the above findings were applicable to native B lymphocytes. We performed live-cell imaging of isolated mouse spleen B lymphocytes and evaluated chemotaxis toward CXCL12 (Fig. 6a–c), in comparison with T lymphocytes (Fig. 6d–f). In both B and T lymphocytes isolated from mouse spleen, the CXCL12 gradient induced chemotaxis (Fig. 6a,b,d,e). Blocking NCLX with 2 μM CGP-37157 significantly suppressed B lymphocyte chemotaxis and motility in the presence of CXCL12 (Fig. 6a–c). These results suggested that NCLX had important roles in the chemotaxis of mouse spleen B lymphocytes, similar to B lymphocyte cell lines. In contrast, CGP-37157 did not affect the chemotaxis or motility of T lymphocytes (Fig. 6d–f), indicating that the contribution of NCLX to chemotaxis was specific to B lymphocytes.

**Figure 7:**
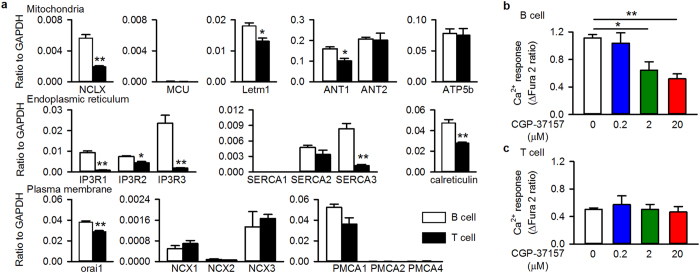
Larger contribution of NCLX on cytosolic Ca^2+^ handling in spleen B lymphocytes than in T lymphocytes. (**a**) mRNA expression of major Ca^2+^ handling proteins expressed relative to GAPDH in mouse spleen B lymphocytes (white bars) and T lymphocytes (black bars). N = 3. (**b**,**c**) Effects of CGP-37157 on responses of cytosolic Ca^2+^ to antigen receptor stimulation by an anti-IgM antibody (10 μg/ml) and an anti-CD3/CD28 antibody (10 μg/ml) in mouse spleen B lymphocytes (**b**) and T lymphocytes (**c**), respectively. The effect of the stimulation on cytosolic Ca^2+^ was evaluated as difference between basal and peak Fura 2 ratio. N = 3–4. Data are expressed as mean ± SEM. **P < 0.01, *P < *0.05.*

NCLX-mediated mitochondria-ER Ca^2+^ recycling is pivotal for the cytosolic Ca^2+^ response to B cell receptor stimulation^20^ and NCLX-mediated regulation of cytosolic Ca^2+^ is associated with the motility of B lymphocytes (Fig. 3, Supplementary Fig. S4). These facts prompted us to compare B and T lymphocytes in terms of their expression of Ca^2+^ carriers and a Ca^2+^ binding protein that determine cytosolic Ca^2+^ dynamics (Fig. 7a). Interestingly, the mRNA levels of factors involved in mitochondria-ER Ca^2+^ dynamics, such as NCLX, the possible mitochondrial H^+^/Ca^2+^ exchanger Letm1, the ER Ca^2+^ pump SERCA3, three ER inositol trisphosphate receptor Ca^2+^ release channels (IP3Rs 1–3), and the ER Ca^2+^ binding protein calreticulin, were significantly higher in B lymphocytes than in T lymphocytes. It should be noted that the expression levels of mitochondrial adenine nucleotide translocator 2 (ANT2) and ATP synthase subunit ATP5b were comparable between B and T lymphocytes, suggesting that the cellular content of mitochondria is similar in the two cell types. In addition, the expression level of orai1, which mediates Ca^2+^ influx across the plasma membrane in response to ER Ca^2+^ depletion, was also higher in B lymphocytes than in T lymphocytes. On the other hand, the expression levels of other Ca^2+^ carriers in plasma membrane, such as the Na^+^-Ca^2+^ exchangers NCX1–3 and the Ca^2+^ pump PMCA1, were comparable between B and T lymphocytes. These results suggest that the contribution of organelle Ca^2+^ dynamics to cytosolic Ca^2+^ was larger in B lymphocytes than in T lymphocytes. In fact, the cytosolic Ca^2+^ response to antigen receptor stimulation was sensitive to the mitochondrial Na^+^-Ca^2+^ exchange blocker CGP-37157 in B lymphocytes but not in T lymphocytes (Fig. 7b,c). The larger contribution of organelle Ca^2+^ dynamics may be related to the larger contribution of NCLX to chemotaxis in B lymphocytes than in T lymphocytes.

## Discussion

NCLX was identified as a mitochondrial Na^+^-Ca^2+^ exchanger by Palty *et al*.^19^, and there is a rapidly growing literature on the physiological and pathophysiological roles of NCLX in a variety of cell types, including pancreatic β cells, astrocytes, cardiomyocytes, and B lymphocytes^20^,^21^,^22^,^36^,^37^,^38^.

In the present study, we showed that NCLX modulates B lymphocyte motility by regulating cytosolic Ca^2+^, and that this is a minor mechanism in T lymphocytes. Under control conditions, a chemokine induces actin polymerization through Rac1, possibly via increasing cytosolic Ca^2+^ (Figs 3 and 4, Supplementary Figs S4 and S5;^8^,^26^,^27^,^29^), and induces mitochondrial polarization (Fig. 5;^33^,^34^), resulting in increased motility with directionality towards the chemokine. On the other hand, NCLX reduction/inhibition increases cytosolic Ca^2+^ even in the absence of chemokine (Fig. 3 and Supplementary Fig. S4), probably due to increased Ca^2+^ leak from the ER, as we reported previously^20^. This Ca^2+^ increase may activate Rac1 and facilitate actin polymerization in many parts of the cell, resulting in augmentation of random movement (Figs 2 and 4, Supplementary Figs S2 and S5). However, the chemokine may hardly be able to induce the further Rac1 activation or actin polarization needed for chemotaxis under this already disorganized condition (Fig. 4 and Supplementary Fig. S5). Moreover, the failure of mitochondrial polarization in response to a chemokine may lead to impaired chemotaxis of cells with reduced or inhibited NCLX, in spite of the increased random migration. The mechanism underlying the prevention of mitochondrial polarization by NCLX reduction/inhibition remains to be clarified. NCLX, or Ca^2+^ extruded by NCLX, is likely to be important for proper localization of mitochondria in the cell, because silencing NCLX disrupts the structural coordination of mitochondria and the ER, as shown in our previous study^20^. We propose here that CXCL12-CXCR4 chemotaxis signalling is associated with NCLX and/or mitochondrial polarization. However, since cytosolic Ca^2+^ affects a wide range of protein functions, other processes besides those examined in this study might be affected by NCLX reduction/inhibition. Further studies are needed to obtain the comprehensive framework of lymphocyte chemotaxis.

There is a limitation in our siRNA experiments. Since siRNA is known to have off-target effects, experiments with several kinds of siRNA are ideal^39^. The experiments were not durable in our experimental system because of relatively low reductions of NCLX mRNA expression with other four siRNAs tested. However, it is notable that similar results to NCLX siRNA experiments in A20 B lymphocytes were obtained by pharmacological inhibition of NCLX in A20 and spleen B lymphocytes and by NCLX knockout in DT40 B lymphocytes. This fact strongly suggests that the off-target effects is small, if any.

Mitochondrial polarization may have another role: to supply ATP to myosin for contraction^33^. Therefore, the impairment of chemotaxis by NCLX suppression may be caused by an inadequate supply of ATP from the mitochondria to myosin. An increase in cytosolic and mitochondrial Ca^2+^ has been reported to activate mitochondrial aspartate/glutamate carriers and dehydrogenases, respectively, resulting in an increased production of NADH and ATP in mitochondria^40^,^41^. However, the cellular ATP level, measured by luciferase assay, was comparable between siControl cells and siNCLX cells (the ATP content of siNCLX cells without CXCL12 was 102.4 ± 6.5% of siControl cells without CXCL12, N = 4). Moreover, CXCL12 did not change the ATP level in either siControl cells or siNCLX cells (in siControl cells and siNCLX cells, the ATP content in the presence of CXCL12 was 100.7 ± 6.5% and 105.0 ± 4.2% of that in the absence of CXCL12, respectively, N = 4). These data suggest that the impairment of chemotaxis by NCLX reduction/inhibition was not due to impaired ATP supply.

As far as we know, this is the first report which demonstrates the involvement of a mitochondrial Ca^2+^ carrier in the regulation of B lymphocyte chemotaxis. Recent findings suggest that the mitochondrial Ca^2+^ uniporter MCU and its accessory protein MICU play roles in the motility of human endothelial cells and zebrafish blastomeres, respectively^17^,^18^. However, in the present study, the contribution of MCU to the chemotaxis of A20 lymphocytes was small (Fig. 1). Moreover, the mRNA expression levels of MCU in mouse spleen B and T lymphocytes were extremely low (6.4 × 10^−5^ ± 1.5 × 10^−5^ and 3.7 × 10^−5^ ± 1.5 × 10^−5^ in mouse spleen B and T lymphocytes, respectively (normalised to glyceraldehyde-3-phosphate dehydrogenase (GAPDH), N = 3) and 0.002 in A20 B lymphocytes (N = 1)), suggesting that the contribution of MCU to chemotaxis in native lymphocytes, if any, is small.

The most unexpected finding was the difference in NCLX contribution to chemotaxis between B lymphocytes and T lymphocytes (Figs 6 and 7a). The particularly important role of NCLX in B lymphocytes may be attributable to the difference in Ca^2+^ dynamics between B and T lymphocytes, that is, the larger contribution of NCLX-mediated mitochondria-ER Ca^2+^ dynamics to cytosolic Ca^2+^ in B lymphocytes than in T lymphocytes (Fig. 7b,c).

It would be interesting to see if NCLX participates in immune responses, such as antibody production, *in vivo*. However, this would be rather difficult because non-specific effects of the mitochondrial Na^+^-Ca^2+^ exchange blocker CGP-37157 on L-type Ca^2+^ channels, which are important in the heart, nerve and muscle, cannot be excluded^42^, and retention of CGP-37157 in the body is extremely low^43^. The production of complete NCLX knockout mice would facilitate such studies.

## Methods

### Solutions and drugs

Ru360 and BAPTA-AM were purchased from Sigma-Aldrich. CGP-37157 was purchased from Tocris Bioscience. The stock solution was prepared with DMSO, the final concentration of which was 0.01–0.1%.

### Cell culture and transfection

Maintenance of murine A20 B lymphocytes and transfection with siRNA were performed as previously described^20^.

### Transwell chemotaxis assay

Assays were performed using Transwells equipped with polycarbonate membrane filters (8-μm pore; Corning) according to the protocol by Hara-Chikuma *et al*.^44^. The membrane was coated with PBS containing 10 μg/ml fibronectin (Sigma-Aldrich) for 30 min, washed twice, and incubated in RPMI 1640 containing 0.1% BSA for 30 min at 37 °C. The lower chamber was filled with RPMI 1640 + 0.1% BSA in the presence or absence of 100 ng/ml recombinant murine CXCL12 (PeproTech). Cells were starved for 1 hr in serum-free RPMI 1640 with 0.1% BSA, then applied to the upper chamber. The migrated cells were counted after 5 hrs by flow cytometry analysis (FACSCalibur; BD Biosciences), and expressed as a percentage of the input cells. When pharmacological inhibitors were used, cells were pretreated with the inhibitors for 20 min at 37 °C, and the inhibitors were added to both chambers.

### Real-time chemotaxis assay

Assays were performed with a μ-Slide Chemotaxis^3D^ (ibidi GmbH) according to the manufacturer’s instructions. Cells were conjugated with collagen I gel and applied to the observation area in the presence or absence of 100 ng/ml CXCL12 in one side of the reservoir. The chamber was placed in a CO_2_-incubator (Tokai hit) on the stage of a fluorescence microscope (ECLIPSE Ti; Nikon). Transmission images of cells were obtained every 2 min over 8 hrs using a digital CCD camera (ORCA-R2; Hamamatsu Photonics), and analyzed using a particle tracking tool of AQUACOSMOS software (Hamamatsu Photonics), a filtering tool of ImageJ (NIH) and a Chemotaxis and Migration tool (ibidi GmbH). Cells which moved directionally toward CXCL12 were evaluated by the position in the 90-degree area facing CXCL12 at the end of the experiment. The extent of chemotaxis was expressed as the percentage of directionally moved cells which moved over a distance equal to the average cell size (A20, ≥15 μm; splenocytes, ≥10 μm). When pharmacological inhibitors were used, they were applied to both sides of the reservoir.

### Measurement of Ca^2+^_i_ in single cells

Cytosolic Ca^2+^ was measured as described previously^20^. Cells which were stimulated with or without 100 ng/ml CXCL12 for 2 hrs were loaded with 5 μM Fura 2-AM, and transferred to a cover glass coated with fibronectin. The fluorescence images of the cells were recorded using an EM-CCD camera (ImagEM, Hamamatsu Photonics) mounted on a fluorescence microscope (ECLIPSE Ti) and analyzed with AQUACOSMOS software.

### F-actin polymerization assay

Cells were applied to cover glasses coated with 10 μg/ml fibronectin, starved for 1 hr at 37 °C in serum-free RPMI 1640 with 0.1% BSA, and incubated with or without 100 ng/ml CXCL12 for 2 hrs. After fixation with 3.7% formalin for 15 min at 37 °C, cells were permeabilised with 0.1% Triton-X/PBS for 10 min, blocked with 1% BSA/PBS for 30 min, and stained with Alexa Fluor 488 phalloidin (Invitrogen) and DAPI (Dojindo). Immunofluorescence images were obtained using a confocal microscope (LSM 710; Zeiss). A high intensity phalloidin signal was defined as a fluorescence intensity of more than 5000 a.i. and was expressed as % of cell area.

### Analysis of mitochondrial polarization

Cells were applied to cover glasses coated with 10 μg/ml fibronectin, starved for 1 hr at 37 °C in serum-free RPMI 1640 with 0.1% BSA, and incubated with or without 100 ng/ml CXCL12 for 2 hrs. Then cells were stained with 1 μM MitoTracker Orange (Invitrogen), and images were obtained using a confocal microscope (LSM 710; Zeiss). Mitochondria-polarized cells were defined as cells in which the mitochondria were located within one-half of the cell area.

### Isolation of mouse spleen B and T lymphocytes

Female 6–10-wk-old Balb/c mice were purchased from Japan SLC. All experimental procedures were conducted in accordance with the guidelines of the Research Center of Kyoto University Graduate School of Medicine and Regulations for Animal Research at University of Fukui. Procedures were approved by the Committee on Animal Research of Kyoto University Graduate School of Medicine and the Faculty of Medical Sciences, University of Fukui. Single splenocytes were obtained by standard procedures^45^, and then B and T lymphocytes were isolated by negative selection using the MACS system (B cell isolation kit (#130–090–862) and CD4^+^ T cell kit (#130–095–248); Miltenyi Biotec K.K.), according to the manufacturer’s instructions.

### Quantitative PCR analysis

Quantitative PCR analysis was performed as described previously^20^,^22^. The primers are listed in Supplementary Table S1.

### Statistical analysis

All data are presented as mean ± SEM of independent recordings. The number of independent experiments and the number of cells per recording are presented as N and n, respectively. In the microscopy measurements, the responses from n individual cells were averaged for each recording. Then the statistical evaluation was performed on averaged responses from N independent recordings. Each experiment was repeated independently more than three times. Statistical analyses were performed by one-way ANOVA multiple comparisons (SigmaPlot, Systat Software Inc., San Jose, CA, USA). Post hoc and two-group comparisons were performed using the Student–Newman–Keuls test and the unpaired Student’s *t* test, respectively. *P* < 0.05 was considered significant.

## Additional Information

**How to cite this article**: Kim, B. *et al*. Roles of the mitochondrial Na^+^-Ca^2+^ exchanger, NCLX, in B lymphocyte chemotaxis. *Sci. Rep.* **6**, 28378; doi: 10.1038/srep28378 (2016).

## Supplementary Material

Supplementary Information
