# Supplementary material for: Roles of the mitochondrial Na+-Ca2+ exchanger, NCLX, in B lymphocyte chemotaxis
Source: Sci Rep. 2016 Jun 22;6:28378. doi: 10.1038/srep28378 (PMC4916421; doi:10.1038/srep28378)
Supplement: Supplementary Information [file srep28378-s1.pdf]

SUPPLEMENTARY INFORMATION

Roles of the mitochondrial Na^{+}-Ca^{2+} exchanger, NCLX, in B lymphocyte chemotaxis

Bongju Kim[1,a], Ayako Takeuchi[2,a], Masaki Hikida[1] and Satoshi Matsuoka[1,2,*]

[1]Center for Innovation in Immunoregulative Technology and Therapeutics, Graduate School of Medicine, Kyoto University, Yoshida-konoe, Sakyo-ku, Kyoto 606-8501, Japan

[2]Department of Integrative and Systems Physiology, Faculty of Medical Sciences, University of Fukui, 23-3 Matsuokashimoaizuki, Eiheiji-cho, Yoshida-gun, Fukui 910-1193, Japan

[a]B.K. and A.T. contributed equally to this work.

Present address of B.K.

Clinical Dental Research Institute, Seoul National University Dental Hospital, Seoul 03080, Korea (email:P6032@snudh.org)

Correspondence and requests for materials should be addressed to S.M. (email: smatsuok@u-fukui.ac.jp).

SI Table

Table S1. Primers used for real time PCR analysis.

clone name	sequence	position	product (bp)	accession No.
**NCLX**				
sense primer	TCGCTGTGACTTTGTCAGGA	338-357	136	NM_133221
antisense primer	AAGCAGCCAGAAAACGTAGAGG	473-452		
**MCU**				
sense primer	GAGCCGCATATTGCAGTACGGT	204-225	149	NM_001033259
antisense primer	AAACACGCCGACTGAGTCAGAG	352-331		
**Letm1**				
sense primer	TCCTGCGTTTCCAGCTCACCAT	1177-1198	149	NM_019694
antisense primer	GTCTTCTGTGACACCGAGAGCT	1325-1304		
**ANT1**				
sense primer	GCTGCCTACTTCGGAGTCTATG	652-673	132	NM_007450
antisense primer	GTCAAACGGATAGGACACCAGC	783-762		
**ANT2**				
sense primer	ACACGGTTCGCCGTCGTATGAT	774-795	115	NM_007451
antisense primer	AAAGCCTTGCTCCCTTCATCGC	888-867		
**ATP5b**				
sense primer	CTCTGACTGGTTTGACCGTTGC	868-889	148	NM_016774
antisense primer	TGGTAGCCTACAGCAGAAGGGA	1015-994		
**IP3R1**				

sense primer	CGATGACATCGTTCGTGTGGTC	4556-4577	100	NM_010585
antisense primer	CACCTCCGTATCCACATAGCAG	4655-4634		
**IP3R2**				
sense primer	CTCTTGCCTCTGGACGACATAG	4543-4564	114	NM_019923
antisense primer	CTCCACTTCAGTGTCAACGTAGC	4656-4634		
**IP3R3**				
sense primer	GCAACCACATCTGGACGCTCTT	4583-4604	138	NM_080553
antisense primer	AGAAGGCACTGATGGTGTCCAG	4720-4699		
**SERCA1**				
sense primer	GAAGCCTCTCTAAAGTGGAGCG	1530-1551	136	NM_007504
antisense primer	CGTGAGGACTTAGCTGGTGAAC	1665-1644		
**SERCA2**				
sense primer	GTGAAGTGCCATCAGTATGACGG	1744-1766	134	NM_009722
antisense primer	GTGAGAGCAGTCTCGGTAGCTT	1877-1856		
**SERCA3**				
sense primer	TGCGGAAAGAGTTCACCCTGGA	1621-1642	137	NM_016745
antisense primer	GCGCTCAATTACACTCTCAGGAG	1757-1735		
**calreticulin**				
sense primer	AAAGGACCCTGATGCTGCCAAG	757-778	108	NM_007591
antisense primer	TCAGGGATGTGCTCTGGCTTGT	864-843		

**orai1**				
sense primer	GGGACGCTGCTTTTCCTAGC	737-756	126	NM_175423
antisense primer	GTGGTTGGCGACGATGACTG	862-843		
**NCX1**				
sense primer	GTGGTGAACTGCCTCCAGAGA	3636-3656	79	NM_011406
antisense primer	CCTTAAACTATGTCTAGAACCATTGCTTT	3714-3686		
**NCX2**				
sense primer	GGAGCATCTTTGCCTATGTCTGG	749-771	133	BC058704
antisense primer	TTGTCCGCCATCCAGGCAAACA	881-860		
**NCX3**				
sense primer	GGACCAGTTCATGGAAGCCATC	2680-2701	140	NM_001167920
antisense primer	CACAGGCAAAGAGCACCTTCCA	2819-2798		
**PMCA1**				
sense primer	GCACAGTCTCAGAGCAACGACA	2791-2812	118	NM_026482
antisense primer	GCCACATCAGTTCCAGCAATGC	2908-2887		
**PMCA2**				
sense primer	CGGATAAGCACACGCTGGTCAA	2874-2895	123	NM_009723
antisense primer	AGCCCACATCTGCCTTCTTGAG	2996-2975		
**PMCA4**				
sense primer	CGTCTTTGCTGGCGACACAC	3271-3290	150	NM_001167949
antisense primer	TCACCGTGGATCTTCCGAGC	3420-3401		

**GAPDH**				
sense primer	TGTGTCCGTCGTGGATCTGA	761-780	77	NM_008084
antisense primer	CCTGCTTCACCACCTTCTTGA	837-817		

SI Materials and Methods

Cell culture

Maintenance of chicken DT40 B lymphocytes and heterozygous NCLX knockout DT40 B lymphocytes (NCLX$^{+/-}$) was performed as previously described[1].

Transwell chemotaxis assay using DT40 B lymphocytes

The Transwell chemotaxis assay was performed similarly to experiments with A20 cells (see Methods), except for the membrane filter (5-μm pore) and CXCL12 (human, PeproTech).

Analysis of surface expression of the CXCL12 receptor CXCR4

A20 B lymphocytes were labeled with or without FITC-conjugated rat anti-mouse CXCR4 (CD184) antibody (BD Pharmingen). The fluorescence was analyzed by flow cytometry (FACSCalibur). Data acquisition and analysis were performed with CellQuest (BD Biosciences) and FlowJo software (Tree Star, Inc.), respectively.

Analysis of Rac1 localization

A20 B lymphocytes were applied to cover glasses coated with 10 μg/ml fibronectin, starved for 1 hr at 37°C in serum-free RPMI 1640 with 0.1% BSA, and incubated with or without 100 ng/ml CXCL12 for 2 hrs. After fixation with 4% paraformaldehyde for 30 min at room temperature, cells were permeabilized with 0.5% Triton-X/PBS for 10 min, blocked with 1% BSA/PBS for 30 min, and incubated with anti-Rac1 monoclonal antibody (Cytoskeleton, Inc.). Then cells were stained with rhodamine-conjugated anti-mouse antibody (Jackson Labs, Inc.) and DAPI (Dojindo). Immunofluorescence images were obtained using a confocal microscope (LSM 710; Zeiss).

Measurement of cellular ATP

A20 B lymphocytes were seeded onto 96-well plates coated with fibronectin, starved for 1 hr at 37°C in serum-free RPMI 1640 with 0.1% BSA, and stimulated with or

without 100 ng/ml CXCL12 for 2 hrs. Cellular ATP level was measured using the CellTiter-Glo Luminescent Cell Viability Assay (Promega K.K.), according to the manufacturer’s instructions.

Analysis of mitochondrial polarization using DT40 B lymphocytes

Cells were applied to cover glasses coated with 10 μg/ml fibronectin, starved for 1 hr at 37°C in serum-free RPMI 1640 with 0.1% BSA, and incubated with or without 100 ng/ml CXCL12 for 2 hrs. Then cells were stained with 200 nM MitoTracker Orange (Invitrogen), and images were obtained using a confocal microscope (TCS SP II; Leica). Mitochondria-polarized cells were defined as cells in which the mitochondria were located within one-half of the cell area.

SI Reference

1. Kim, B. *et al.* Pivotal role of mitochondrial Na^+-Ca^{2+} exchange in antigen receptor mediated Ca^{2+} signalling in DT40 and A20 B lymphocytes. *J Physiol* **590**, 459-474 (2012).

SI Figure legends

Fig. S1. Effects of silencing NCLX on CXCL12-induced chemotaxis of DT40 B lymphocytes evaluated by Transwell assay.

WT, wild type DT40 B lymphocytes; $NCLX^{+/-}$, NCLX heterozygous knockout DT40 B lymphocytes. N = 3. CXCL12 (100 ng/ml) was applied to the lower chamber. Data are expressed as mean ± SEM. $^{**}P < 0.01$.

Fig. S2. Effects of NCLX inhibition and hetero knockout on CXCL12-induced cell movement in A20 B lymphocytes (a-c) and DT40 B lymphocytes (d, e).

(a-c) Effects of the mitochondrial Na^+-Ca^{2+} exchange inhibitor CGP-37157 on the chemotaxis of A20 B lymphocytes in a real-time chemotaxis assay. **(a)** Representative data for cell trajectory. CXCL12 (100 ng/ml) was applied to the left side of the reservoir, and chemotaxis was observed in the absence or presence of CGP-37157 (2 μM) in both sides of the reservoir. **(b)** Percentage of cells which moved toward CXCL12. **(c)** Mean displacement of cells. N = 4–5. **(d,e)** Effects of NCLX heterozygous knockout on the cell migration of DT40 B lymphocytes. CXCL12 (100 ng/ml) was

applied to the cells on the cover glass. **(d)** Representative data for cell trajectory. **(e)** Velocity of cells. N = 3. Data are expressed as mean ± SEM. WT, wild type DT40 B lymphocytes; NCLX$^{+/-}$, NCLX heterozygous knockout DT40 B lymphocytes. $^{**}P$ < 0.01, $^{*}P$ < 0.05, n.s. not significant.

Fig. S3. Effects of NCLX knockdown on expression of the surface chemokine receptor CXCR4.

The surface chemokine receptor CXCR4 (CD184) was evaluated by labelling cells without (dotted lines) or with (solid lines) FITC-conjugated rat anti-mouse CXCR4 (CD184). siControl, control siRNA transfected cells; siNCLX, NCLX siRNA transfected cells.

Fig. S4. Effects of the mitochondrial Na^{+}-Ca^{2+} exchange inhibitor CGP-37157 on cytosolic Ca^{2+}.

A20 B lymphocytes were incubated in the absence (white bars) or presence (black bars) of CXCL12 (100 ng/ml), and cytosolic Ca^{2+} was evaluated by staining the cells with Fura 2-AM (5 μM) 2 hrs after the CXCL12 application. The concentrations of CGP-37157 in μM are shown. N = 14. Data are expressed as mean ± SEM. $^{**}P$ < 0.01, $^{*}P$ < 0.05, n.s. not significant.

Fig. S5. Effects of NCLX knockdown on Rac1 localization.

Rac1 localization was examined by immunocytochemistry. **(a)** Representative data. Rac1 (red) and nuclei (blue) are shown. Right panels show 3D surface plots of fluorescence intensity of rhodamine-conjugated antibody to Rac1 monoclonal antibody. **(b)** Summary. N = 19–20. siControl, control siRNA transfected cells; siNCLX, NCLX siRNA transfected cells. Data are expressed as mean ± SEM. $^{**}P$ < 0.01.

Fig. S6. Attenuation of CXCL12-induced mitochondrial polarization in NCLX$^{+/-}$ DT40 B lymphocytes

Effects of NCLX heterozygous knockout on mitochondrial polarization in the absence or presence of 100 ng/ml CXCL12, evaluated by staining the cells with a mitochondria specific dye (MitoTracker Orange, red). **(a)** Representative data. **(b)** Summary. N = 15. Data are expressed as mean ± SEM. WT, wild type DT40 B

lymphocytes; NCLX$^{+/-}$, NCLX heterozygous knockout DT40 B lymphocytes. *$P < 0.05$, n.s. not significant.

Fig. S1

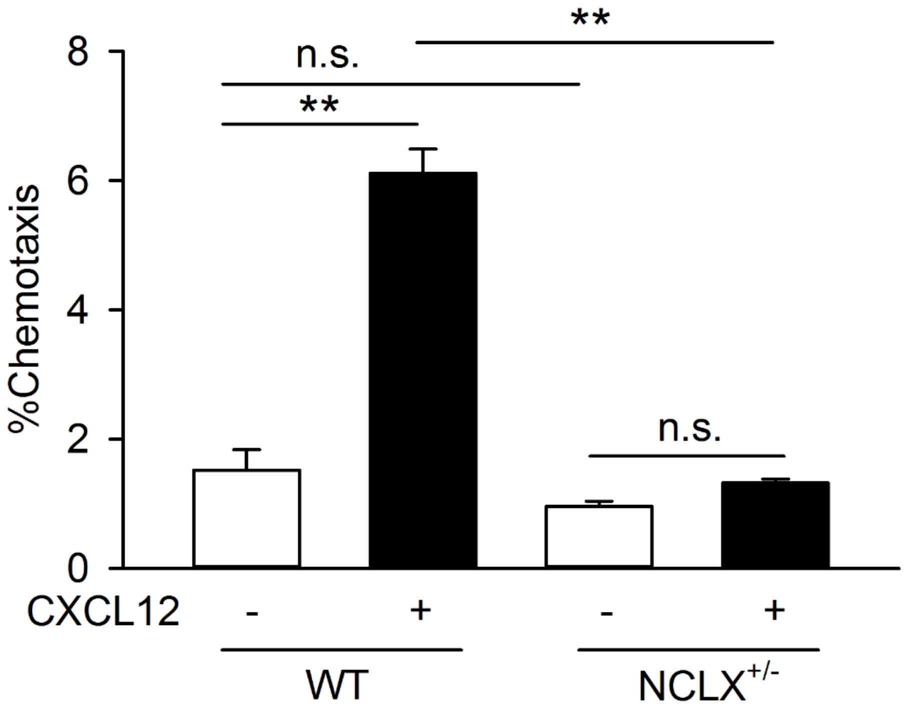

Fig. S2

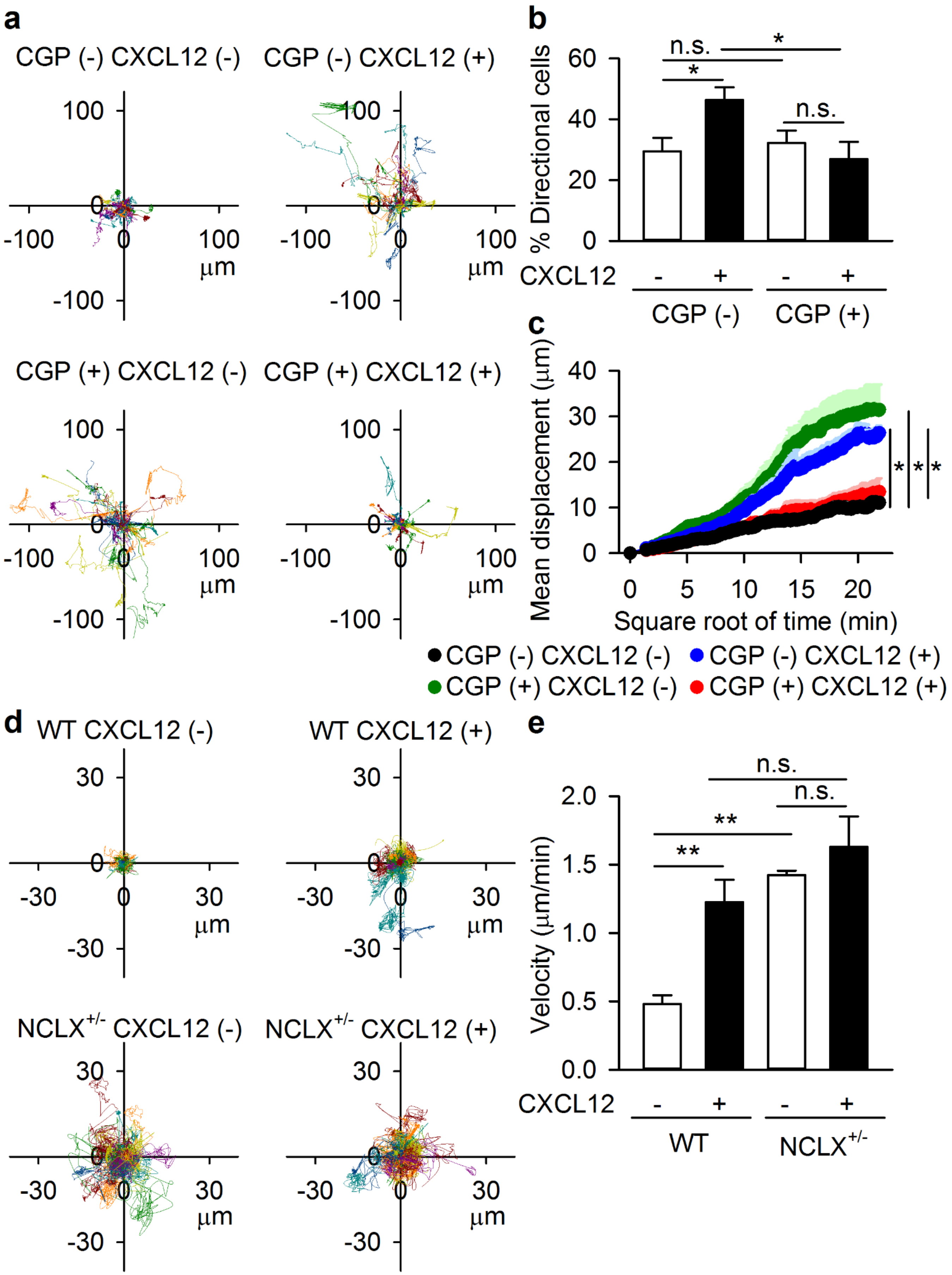
a
CGP (-) CXCL12 (-)
CGP (-) CXCL12 (+)
CGP (+) CXCL12 (-)
CGP (+) CXCL12 (+)
100
0
-100
µm
b
% Directional cells
0
20
40
60
n.s.
*
*
n.s.
CXCL12
-
+
CGP (-)
CGP (+)
c
Mean displacement (µm)
0
10
20
30
40
0
5
10
15
20
Square root of time (min)
*
*
*
CGP (-) CXCL12 (-)
CGP (-) CXCL12 (+)
CGP (+) CXCL12 (-)
CGP (+) CXCL12 (+)
d
WT CXCL12 (-)
WT CXCL12 (+)
NCLX+/- CXCL12 (-)
NCLX+/- CXCL12 (+)
30
0
-30
µm
e
Velocity (µm/min)
0.0
0.5
1.0
1.5
2.0
n.s.
n.s.
**
**
CXCL12
-
+
WT
NCLX+/-

Fig. S3

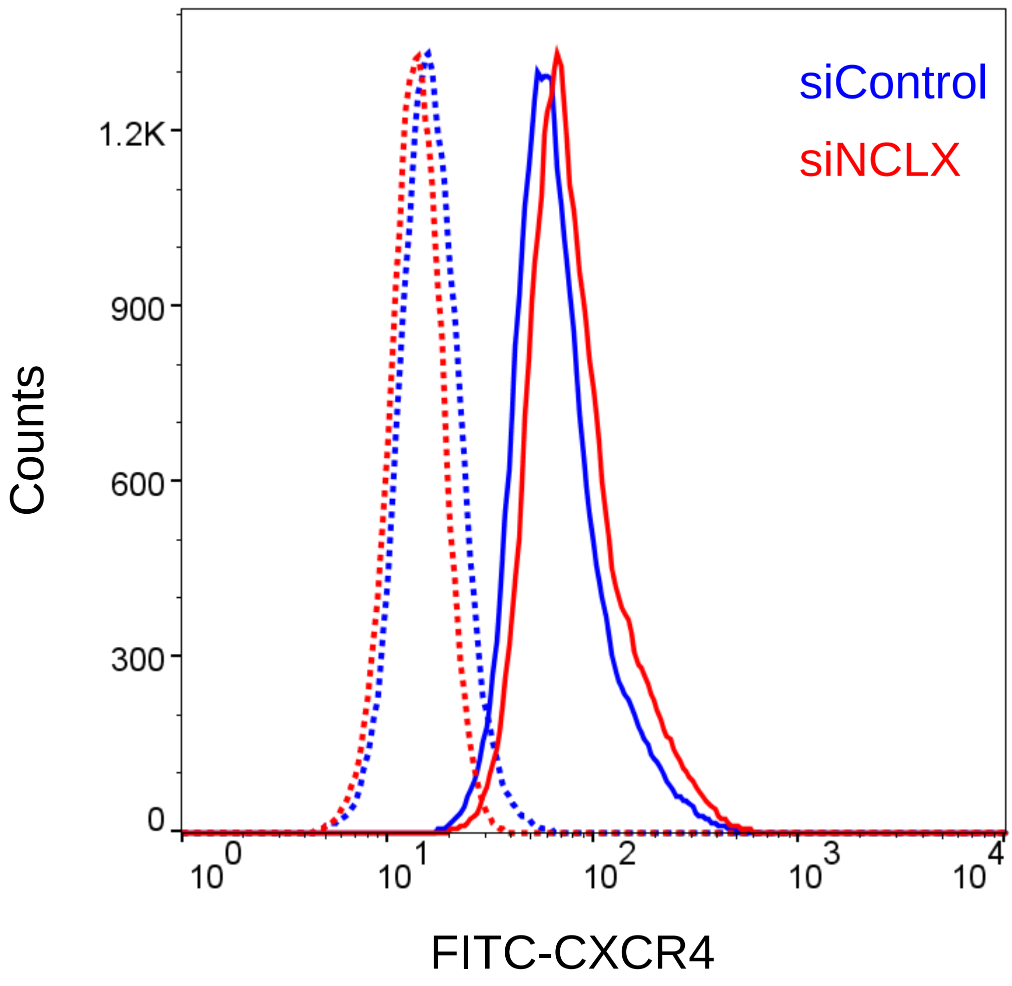

siControl
siNCLX
1.2K
900
600
300
0
Counts
10^0
10^1
10^2
10^3
10^4
FITC-CXCR4

Fig. S4

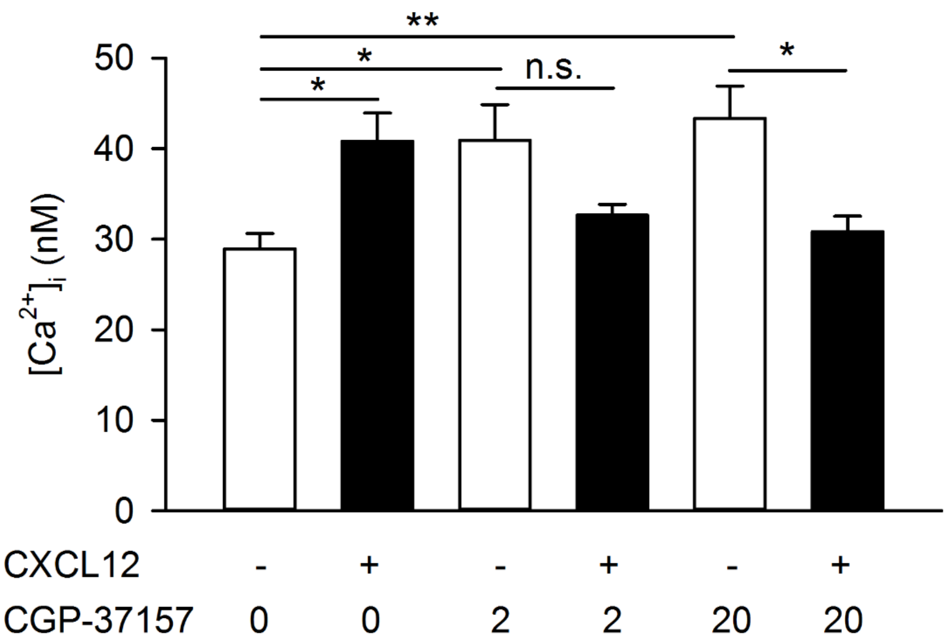

Fig. S5

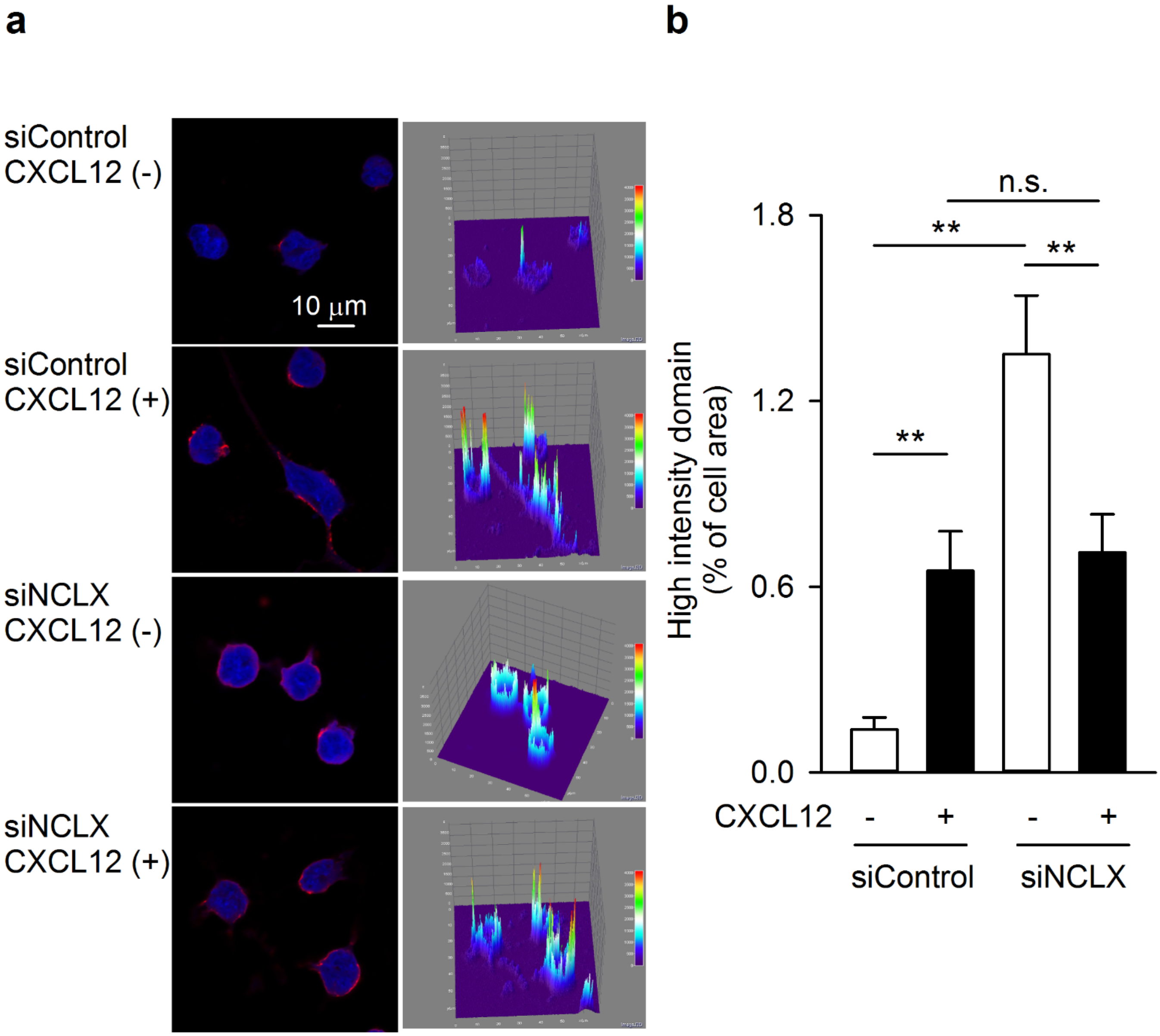
a
siControl
CXCL12 (-)
10 μm
siControl
CXCL12 (+)
siNCLX
CXCL12 (-)
siNCLX
CXCL12 (+)
b
High intensity domain
(% of cell area)
1.8
1.2
0.6
0.0
n.s.
**
**
**
CXCL12
-
+
-
+
siControl
siNCLX

Fig. S6

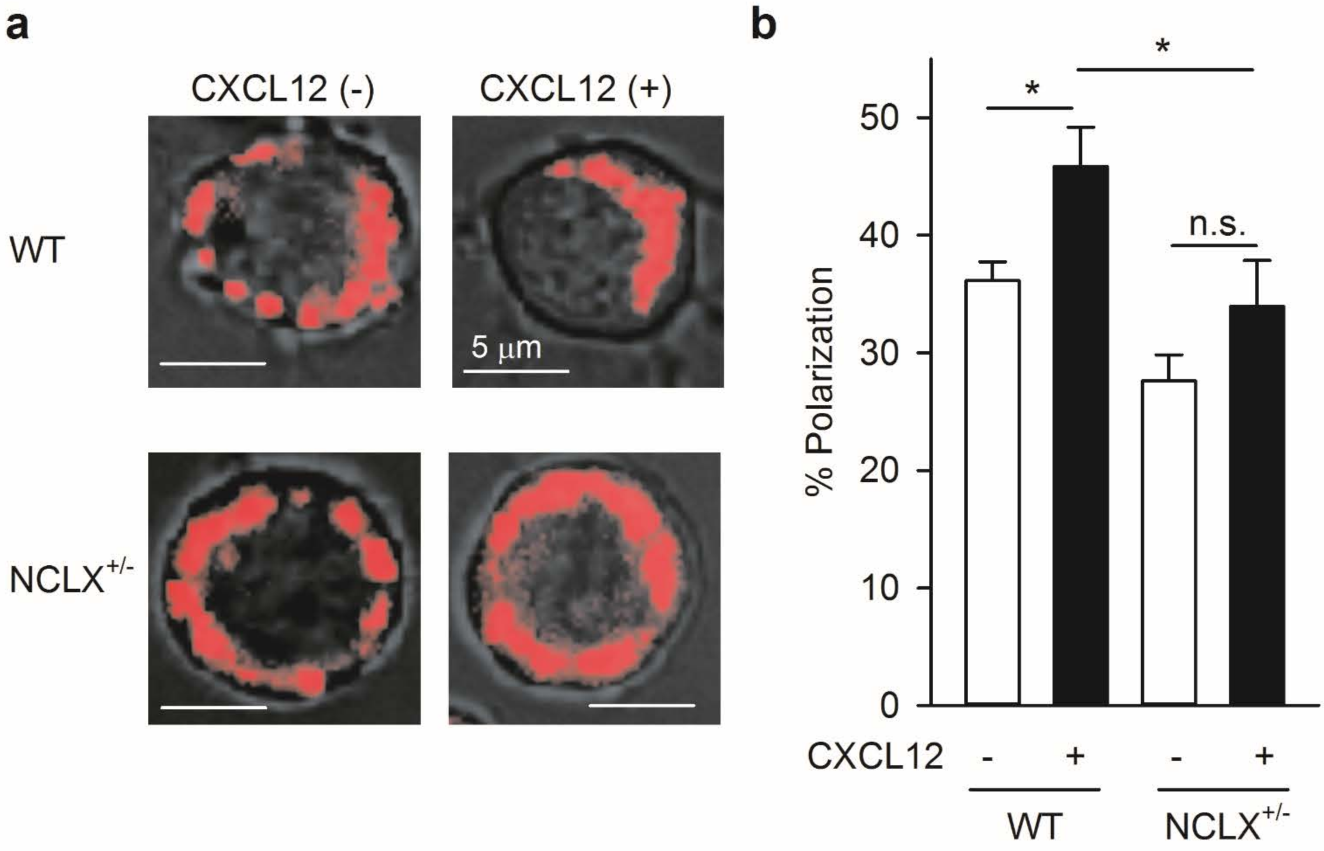
a
CXCL12 (-)
CXCL12 (+)
WT
NCLX+/-
5 μm
b
*
*
n.s.
50
40
30
20
10
0
% Polarization
CXCL12
-
+
-
+
WT
NCLX+/-
